# Low-dose 2-deoxyglucose and metformin synergically inhibit proliferation of human polycystic kidney cells by modulating glucose metabolism

**DOI:** 10.1038/s41420-019-0156-8

**Published:** 2019-03-11

**Authors:** Jing Zhao, Yuxiang Ma, Yingjie Zhang, Bo Fu, Xiaoyuan Wu, Qinggang Li, Guangyan Cai, Xiangmei Chen, Xue-Yuan Bai

**Affiliations:** Department of Nephrology, Chinese PLA General Hospital, Chinese PLA Institute of Nephrology, State Key Laboratory of Kidney Diseases, National Clinical Research Center for Kidney Diseases, Beijing Key Laboratory of Kidney Diseases, Beijing, 100853 China

## Abstract

Polycystic kidney disease (PKD) is a common hereditary kidney disease with abnormal proliferation and apoptosis of kidney cystic epithelial cells, eventually leading to chronic renal failure. Currently, there are no effective treatment methods. Similar to tumor cells, cystic epithelial cells have abnormal glycolysis and over-activation of proliferation signaling pathways. In the present study, for the first time, we investigated the effects of low-dose combinational use of 2-deoxyglucose (2-DG) and metformin (MET) on the proliferation and apoptosis in the human cystic kidney epithelial cells. Cystic epithelia cells were divided into control group, 2-DG group, MET group and 2-DG+MET group. Cell Proliferation, apoptosis and glucose metabolism were measured in each group. The results showed that low-dose combinational treatment of 2-DG and MET significantly inhibited the proliferation of renal cystic epithelial cells by suppressing the activities of PKA, mTOR and ERK signaling pathways and upregulating PI3K/Akt pathway. Combination of both drugs increased the apoptosis rates of cystic epithelial cells. Two drugs inhibited glucose metabolic phenotypes, glycolysis and oxidative phosphorylation, and significantly lowered the intracellular ATP level in cystic epithelial cells. 2-DG could also neutralize excessive production of lactate (lactic acidosis) caused by MET and both drugs had complementary effect for cystic epithelial cells. These results reveal that combinational use of low-dose 2-DG and MET can markedly inhibit proliferation via modulating glucose metabolic phenotypes in human polycystic kidney epithelial cells, low-dose combinational use of both drugs can also lower the toxic effects of each drug, and is a novel strategy for future treatment of human polycystic kidney disease.

## Introduction

Polycystic kidney disease (PKD) is a hereditary kidney disease. Both kidneys in PKD are filled with multiple serous cysts derived from renal tubules; the cyst epithelial cells show abnormal proliferation and gradually increase in volume, thus compressing normal kidney tissues and eventually leading to end-stage kidney disease^1^. The pathogenesis of PKD is still unclear, and there is no effective treatment.

In recent years, the Warburg effect has been found in polycystic kidney epithelial cells, similar to tumor cells. Under aerobic conditions, the cystic cells mainly rely on glycolytic metabolism for energy supply rather than on mitochondrial oxidative phosphorylation^2,3^. Additionally, the activity of the energy sensor, adenosine monophosphate activated protein kinase (AMPK), is decreased, while the mammalian target of rapamycin (mTOR) signaling pathway is over-activated in cyst epithelial cells^4,5^. Furthermore, the proliferation-related signaling pathways, cyclic adenyl-monophosphate-protein kinase A (cAMP-PKA) and extracellular-regulated protein kinase (ERK), are activated, while the activity of phosphoinositide 3-kinase (PI3K)/Akt signaling pathway that inhibits the over-activation of ERK proliferation signaling pathway is significantly inhibited in the cystic cells^6^.

Numerous anti-proliferative drugs, such as rapamycin (mTOR inhibitor) and octreotide (somatostatin analog), have been used to treat polycystic kidney animal models in recent years. Although these drugs showed good efficacy in cells and animal models, the effects were not satisfactory in a number of follow-up clinical trials^7^. Tolvaptan, a vasopressin V2 receptor antagonist, is also effective; however, clinical studies have shown that patients suffer severe thirst, polyuria, nocturia, polydipsia and liver toxicity, and the US Food and Drug Administration (FDA) has not yet approved this drug for clinical use^8^. Therefore, there is an urgent need to find new treatment methods.

2-Deoxyglucose (2-DG) is a glucose analog that inhibits glycolysis^9,10^. 2-DG can compete with glucose to bind hexokinase (the first rate-limiting enzyme of glycolysis) in cells and inhibit metabolism of tumor cell, thereby inhibiting cell proliferation^11^. Metformin (MET) is a first-line drug for the clinical treatment of type 2 diabetes mellitus. Recent studies have found that MET can specifically inhibit mitochondrial respiratory chain complex I and decrease oxidative phosphorylation levels in cells, thus reducing adenosine triphosphate (ATP) synthesis, activating AMPK and inhibiting mTOR proliferation signaling pathway^12–16^. Due to the obvious activation of glycolysis in tumor cells, a large quantity of glucose is consumed and high levels of ATP are produced, resulting in a decrease in AMP/ATP ratio and significantly inhibited AMPK activity^17^. Thus, glycolytic inhibitor 2-DG and AMPK activator MET have been used in the treatment of tumors in recent years. The combinational use of MET and 2-DG can significantly deplete the ATP supply of cancer cells and inhibit the over-activation of proliferation signaling pathways in cells, thereby significantly inhibiting the over-proliferation of tumor cells and reducing the side effects caused by high doses of the individual drugs^18–20^.

In the present study, for the first time, we treated human polycystic kidney cyst-lining epithelial cells with a combination of low-dose MET and 2-DG. We systematically analyzed the effects of the combination of these two drugs on the proliferation and apoptosis of cyst epithelial cells and explored the possible molecular mechanisms.

## Results

### Combinational use of low-dose 2-DG and MET significantly inhibits the proliferation of human polycystic kidney epithelial cells

**Fig. 1:**
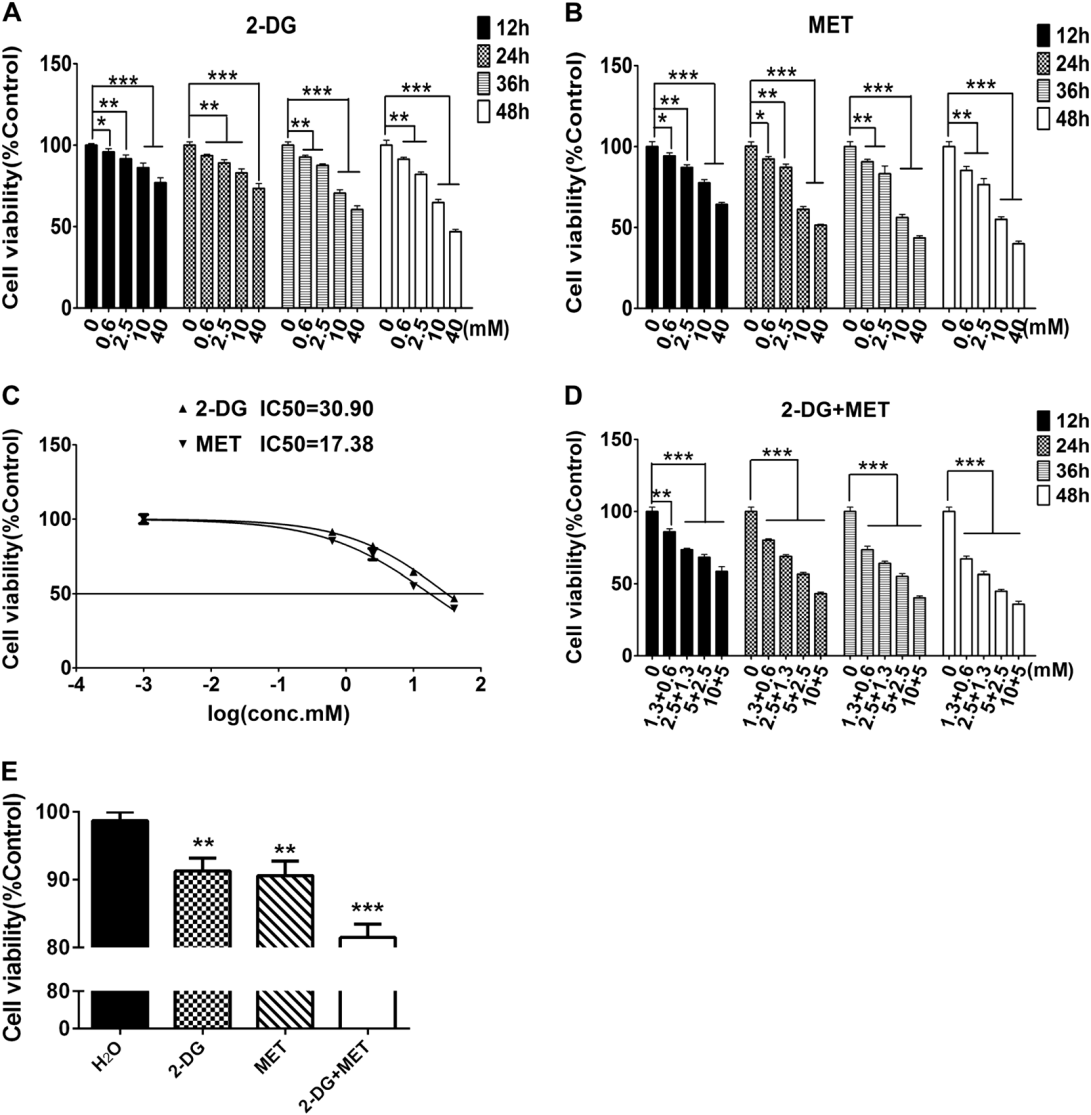
Effects of 2-deoxyglucose (2-DG), metformin (MET) and their combination on the proliferation of WT9-7 cells and human renal proximal tubular epithelial cells (HRPTEC) evaluated using Cell Counting Kit-8 (CCK-8) assay. **a** WT9-7 cells were treated with 2-DG (0.6, 2.5, 10 and 40 mM) for 12, 24, 36 and 48 h, and the cell viability was determined using CCK-8 assay. **b** WT9-7 cells were treated with MET (0.6, 2.5, 10 and 40 mM) for 12, 24, 36 and 48 h, and the cell viability was determined using CCK-8 assay. **c** Cells were treated with various concentrations (0.6, 2.5, 10 and 40 mM) of 2-DG or MET for 48 h, and the half- maximal inhibitory concentration (IC_50_) was determined using GraphPad Prism software. **d** The combined treatment with 2-DG and MET enhanced the growth inhibition of WT9-7 cells. WT9-7 cells were treated with 2-DG and MET at different doses (1.3 + 0.6, 2.5 + 1.3, 5 + 2.5 and 10 + 5 mM) for 12, 24, 36 and 48 h, and the cell viability was determined using the CCK-8 assay. **e** HRPTEC cells were treated with 2-DG (5 mM), MET (2.5 mM) and their combination for 48 h, and the cell viability was determined using CCK-8 assay; *p < 0.05, **p < 0.01, ***p < 0.001 vs Control

The effects of individual 2-DG and MET on cell proliferation were evaluated using a Cell Counting Kit-8 (CCK-8) assay in human polycystic kidney epithelial cells WT9-7 treated with different concentrations (0.6, 2.5, 10 and 40 mM) of 2-DG or MET alone for different times (12, 24, 36 and 48 h). When the cells were treated with different drugs for the same time, the viable cell count gradually declined with increasing concentration of the drugs compared with that in control group. This finding indicates that the inhibitory effects of 2-DG and MET on the proliferation of cystic epithelial cells were dose dependent. When cells were treated with the same drugs for different times, the inhibitory effects of the drugs on cell proliferation were gradually enhanced as the treatment time increased. These results indicate that the inhibitory effects of these drugs were time dependent, and the highest inhibition rate was found at 48 h (Fig. 1a, b, Tables 1 and 2).

**Table 1:** Effect of 2-deoxyglucose (2-DG) on proliferation of polycystic kidney epithelial cells

	0 mM	0.6 mM	2.5 mM	10 mM	40 mM
12 h	100.00 ± 2.36	95.86 ± 1.89	91.61 ± 2.31**	86.22 ± 2.75***	76.92 ± 2.99***
24 h	100.00 ± 2.41	93.58 ± 0.82*	89.13 ± 1.99***	82.88 ± 2.60***	73.43 ± 2.93***
36 h	100.00 ± 2.34	92.66 ± 1.14**	87.58 ± 0.90***	70.55 ± 2.06***	60.48 ± 2.32***
48 h	100.00 ± 2.28	91.40 ± 1.18***	82.00 ± 1.49***	64.75 ± 2.00***	46.88 ± 1.48***

*P < 0.05, **p < 0.01, ***p < 0.001 vs Control

**Table 2:** Effect of metformin (MET) on proliferation of polycystic kidney epithelial cells

	0 mM	0.6 mM	2.5 mM	10 mM	40 mM
12 h	100.00 ± 2.89	94.22 ± 1.91*	87.09 ± 1.70***	77.57 ± 2.02***	64.26 ± 1.17***
24 h	100.00 ± 2.56	92.28 ± 1.61**	87.23 ± 2.00***	61.24 ± 1.73***	51.42 ± 0.57***
36 h	100.00 ± 2.35	90.68 ± 1.47**	83.14 ± 4.90***	56.20 ± 1.92***	43.56 ± 1.31***
48 h	100.00 ± 3.21	85.26 ± 2.56***	76.42 ± 3.73***	55.04 ± 1.59***	39.93 ± 1.55***

*P < 0.05, **p < 0.01, ***p < 0.001 vs Control

The half-maximal inhibitory concentration (IC_50_) for cystic epithelial cells treated with 2-DG or MET alone for 48 h was 30.9 and 17.4 mM, respectively (Fig. 1c). Using the above IC_50_ values, we selected different concentrations (1.3 + 0.6, 2.5 + 1.3, 5 + 2.5 and 10 + 5 mM) of 2-DG+MET to treat cystic epithelial cells for different times (12, 24, 36 and 48 h). The results showed that the combination of the two drugs significantly inhibited the proliferation of cystic epithelial cells. The proliferation inhibition rate reached 50% in cystic epithelial cells treated with the combination of 5 mM 2-DG and 2.5 mM MET for 48 h (Fig. 1d, Table 3). Taking into account the safety of the drugs, we selected small dose of 5 mM and 2.5 mM as the treatment concentrations of 2-DG and MET respectively in the subsequent studies.

**Table 3:** Effects of combined use of 2-deoxyglucose (2-DG) and metformin (MET) on proliferation of polycystic kidney epithelial cells

	0 + 0 mM	1.3 + 0.6 mM	2.5 + 1.3 mM	5 + 2.5 mM	10 + 5 mM
12 h	100.00 ± 2.18	85.92 ± 2.15***	73.47 ± 0.94***	68.16 ± 1.98***	58.40 ± 3.25***
24 h	100.00 ± 3.21	80.02 ± 1.00***	68.83 ± 1.22***	56.53 ± 1.20***	43.21 ± 1.05***
36 h	100.00 ± 1.97	73.43 ± 2.52***	63.98 ± 1.44***	55.11 ± 1.78***	40.31 ± 1.30***
48 h	100.00 ± 2.43	67.02 ± 2.00***	56.41 ± 2.04***	44.87 ± 1.35***	35.81 ± 2.12***

*P < 0.05, **p < 0.01, ***p < 0.001 vs Control

Subsequently, we evaluated the effects of 5 mM 2-DG, 2.5 mM MET and their combination on the viability of HRPTEpiC cells (human renal proximal tubular epithelial cells). The results showed that the effects of 2-DG, MET and their combination on cell viability of HRPTEpiC cells were weaker than on cystic epithelial cells, showing a therapeutic potential of the combination of 2-DG and MET in PKD (Fig. 1e).

**Fig. 2:**
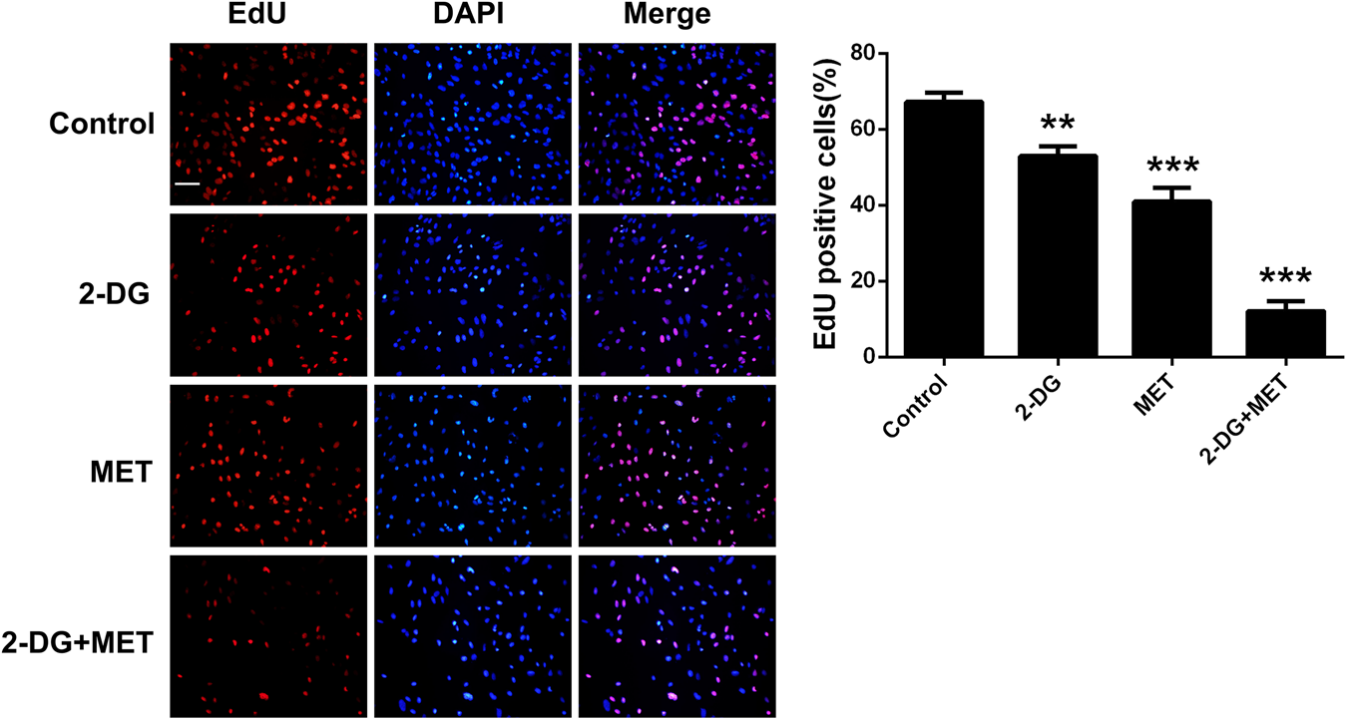
The 5-ethynyl-2'-deoxyuridine (EdU)-positive cells represent new dividing cells and are stained (red). The nucleus was counterstained with 4’,6-diamidino-2-phenylindole (DAPI; blue), original magnification ×100. Scale bar = 15 µm; **p < 0.01, ***p < 0.001 vs Control

CCK-8 assay evaluated cell proliferation mainly through determining the viable cell counts in the plates; however, this method could not fully reflect the proliferation effect of the cells. To further verify whether the combination of 2-DG and MET can significantly inhibit the proliferation of cystic epithelial cells, we directly observed the inhibitory effect of their combination on cell proliferation using 5-ethynyl-2'-deoxyuridine (EdU) nucleic acid labeling technique. The results showed that either 5 mM 2-DG alone or 2.5 mM MET alone, and their combination significantly inhibited the proliferation of cystic epithelial cells (Fig. 2).

### Effects of 2-DG and MET on proliferation-related signaling pathways in human polycystic kidney epithelial cells

A previous study showed that the activation levels of B-Raf/ERK, mTOR and cAMP-PKA proliferation signaling pathways are increased in PKD, while the activation level of PI3K/Akt pathway is decreased^21^. In the present study, we observed the effects of 2-DG or MET alone and combination on the phosphorylation level (representing the activation level) of proliferation signaling pathway molecules in cystic epithelial cells.

**Fig. 3:**
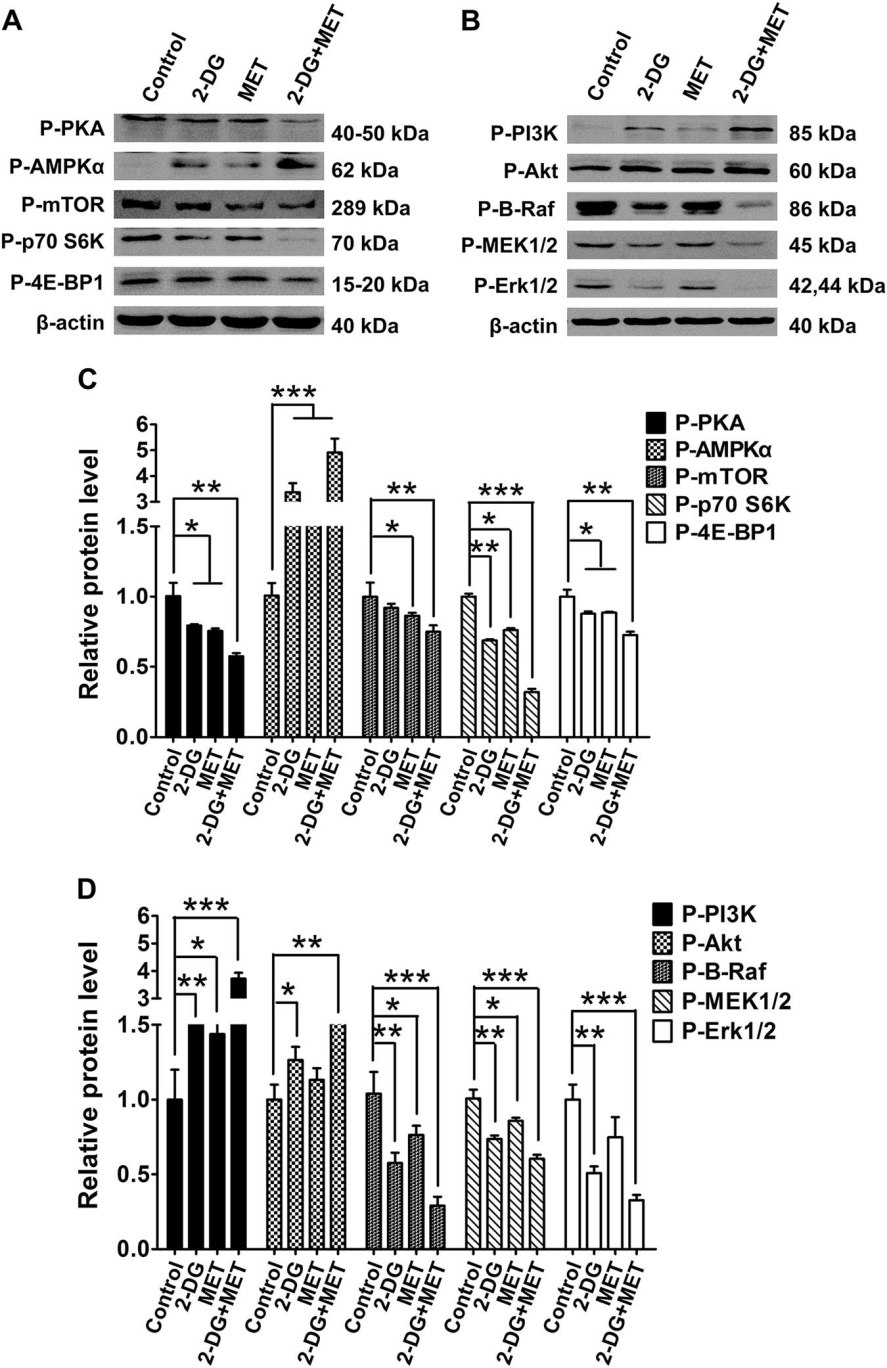
Effects of 2-deoxyglucose (2-DG), metformin (MET) and 2-DG+MET on the B-Raf/ERK, AMPK-mTOR, AKT and PKA signaling pathway molecules in WT9-7 cells. **a**, **b** Protein expression levels of p-PI3K, p-Akt, p-PKA, p-AMPKα, p-mTOR, p-p70 S6K, p-4E-BP1, p-c-Raf, p-MEK1/2 and P-Erk1/2 (p (phospho), represents the protein activation level) were determined using western blotting. **c**, **d** Quantification of relative β-actin protein abundance shown in (**a**, **b**); *p < 0.05,**p < 0.01, ***p < 0.001 vs Control. ERK:  extracellular-regulated protein kinase, AMPK:  adenosine monophosphate activated protein kinase, mTOR:  mammalian target of rapamycin, PKA: protein kinase A

The results showed that, in 2-DG group, the activity of PKA was significantly downregulated; the activity of AMPK was increased; the activating levels of mTOR, p70 S6K and 4E-BP1 were deceased; the activities of PI3K and Akt were markedly upregulated; and the activation levels of B-Raf, MEK1/2, and Erk1/2 were markedly decreased compared with those in control group (Fig. 3).

In MET group, the activity of PKA was significantly downregulated; the activation level of AMPK was increased; the activation levels of mTOR, p70 S6K and 4E-BP1 were decreased; the activation levels of PI3K and Akt were slightly upregulated; and the activation levels of B-Raf, MEK1/2 and Erk1/2 were not significantly decreased compared with those in control group (Fig. 3).

In 2-DG+MET group, the activity of PKA was markedly downregulated; the activation level of AMPK was significantly increased; the activation levels of mTOR, p70 S6K and 4E-BP1 were significantly decreased; the activation levels of PI3K and Akt were markedly upregulated; and the activation levels of B-Raf, MEK1/2 and Erk1/2 were significantly decreased compared with those in control group. These results indicate that the combinational use of these two drugs has synergistic inhibitory effect for the activation of proliferation signaling pathway molecules in cystic epithelial cells (Fig. 3).

### Effects of 2-DG and MET on cell cycle in human polycystic kidney epithelial cells

**Fig. 4:**
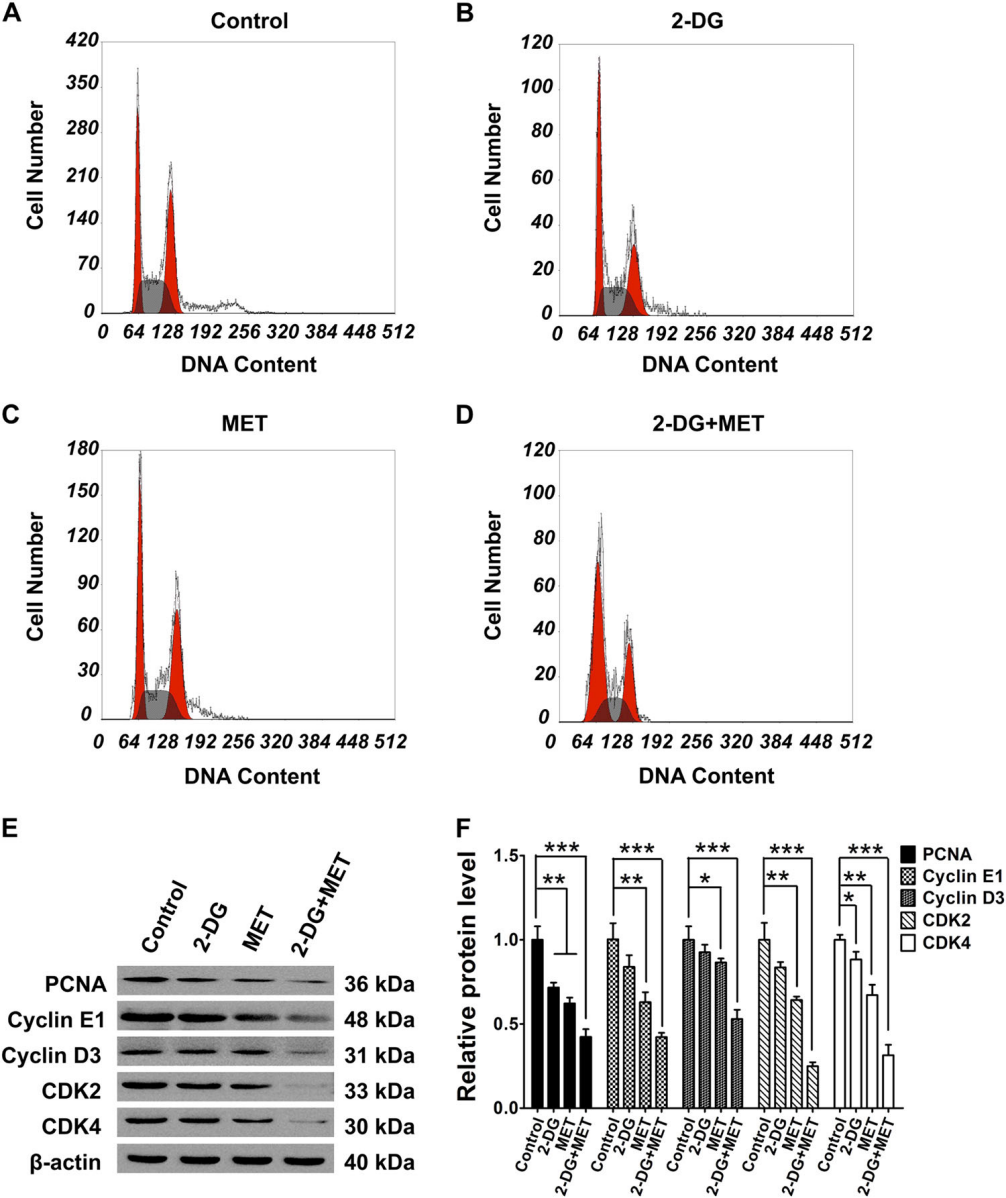
Effects of 2-deoxyglucose (2-DG), metformin (MET) and 2-DG+MET on proliferating cell nuclear antigen (PCNA), the cell cycle and cell cycle proteins in WT9-7 cells. **a–d** Results of cell cycle analysis by flow cytometry (FCM) in WT9-7 cells treated with 5 mM 2-DG, 2.5 mM MET and their combination for 48 h. **e**, **f** Protein expression levels of PCNA, cyclin D3, cyclin E1, CDK2 and CDK4 in WT9-7 cells; *p < 0.05, **p < 0.01, ***p < 0.001 vs Control

Cell proliferation is strongly related to regulation of cell cycle. We analyzed the changes of cell cycle in each drug-treated group by flow cytometry. The results showed that 2-DG or MET alone could extend the G0/G1 phase and reduce the S phase (*p* < 0.05), but the combination of 2-DG and MET markedly extended the G0/G1 phase and reduced the S and G2/M phases (Fig. 4a–d, Table 4) compared with those in control group (*p* < 0.001). These results are indicative of an arrest at the G0/G1 restriction point by a reduction in cell cycle progression. The western blot results showed that the combination of the two drugs markedly decreased the expression of cell cycle proteins, cyclin D3 and cyclin E1, as well as the expression of cyclin-dependent kinases, CDK2 and CDK4 (Fig. 4e, f), indicating that combinational use of the two drugs can significantly arrest cell cycle progression by suppressing the expression of cell cycle proteins.

**Table 4:** The percentage of WT9-7 cells in G0/G1, S, and G2/M phases

Group	G0–G1 (%)	S (%)	G2-M (%)
Control	30.71 ± 1.78	35.27 ± 2.05	34.03 ± 2.48
2-DG	37.64 ± 2.50*	25.76 ± 4.03*	36.60 ± 1.92
MET	38.55 ± 2.45*	28.81 ± 1.06**	33.31 ± 1.98
2-DG+MET	58.16 ± 2.32***	21.36 ± 1.52***	20.48 ± 3.25**

*Control* normal group, *2-DG* 2-deoxyglucose-alone group, *MET* metformin-alone group, *2-DG+MET* the combination of 2-DG and metformin

*P <  0.05, **p <  0.01, ***p <  0.001 vs Control, *n* = 3

Proliferating cell nuclear antigen (PCNA) is present in the nucleus and plays an important role in the initiation of cell proliferation; PCNA is an indicator reflecting the state of cell proliferation. Here, we analyzed the changes of PCNA protein expression in each group by western blot. The results showed that PCNA expression level decreased in all drug-treated groups; however, the effect was more noticeable in 2-DG+MET group than in control group (Fig. 4e, f).

### Effects of 2-DG and MET on apoptosis of human polycystic kidney epithelial cells

**Fig. 5:**
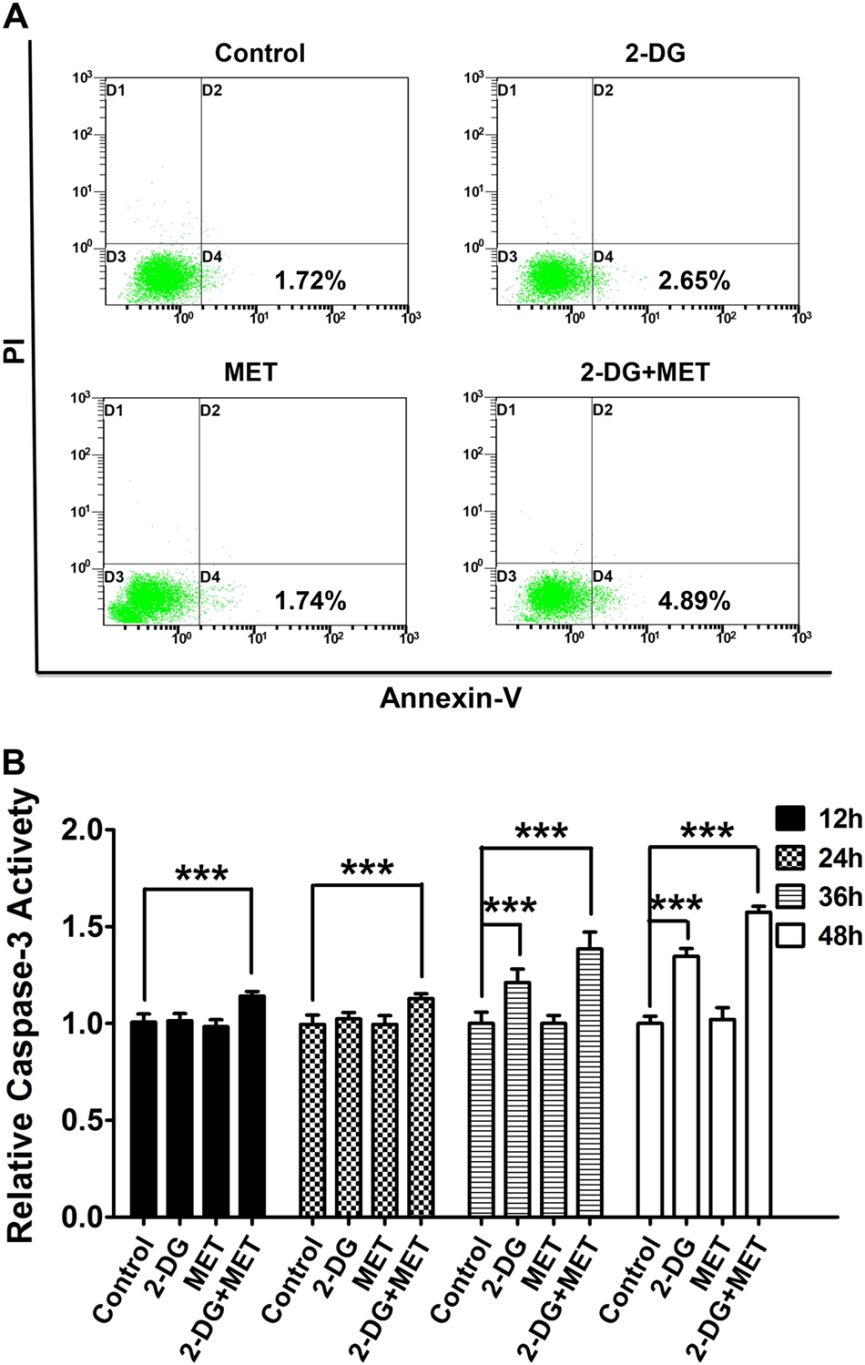
Effects of 2-deoxyglucose (2-DG), metformin (MET) and 2-DG+MET on the apoptosis of WT9-7 cells, as analyzed using annexin V/propidium iodide (PI) staining by flow cytometry (FCM) and a caspase-3 activity assay kit. **a** Results from analyzing the apoptosis rate (D1: necrosis; D2: late apoptosis; D3: living; D4: early apoptosis) by FCM in WT9-7 cells treated with 5 mM 2-DG, 2.5 mM MET and their combination for 48 h. **b** Caspase-3 activity in WT9-7 cells treated with 5 mM 2-DG, 2.5 mM MET and their combination for 12, 24, 36, and 48 h; ***p < 0.001 vs Control

The above results show that the combination of 2-DG and MET markedly decreased the viability of cystic epithelial cells. We hypothesize that the combination of 2-DG and MET can also induce the apoptosis of cystic epithelial cells while inhibiting the proliferation of cystic epithelial cells. To validate this hypothesis, we also tested the effects of 2-DG, MET and their combination on apoptosis. The results showed that the treatment with MET alone had no effect on apoptosis of cystic epithelial cells. However, the intracellular caspase-3 activity increased at 36 h (*p* < 0.001) and the early apoptosis rate also slightly increased at 48 h in the cystic epithelial cells treated with 2-DG alone. The combination of 2-DG and MET significantly promoted the apoptosis of the cystic epithelial cells, resulting in significant increases in the intracellular caspase-3 activity (*p* < 0.001) and early apoptosis rate (Fig. 5, Table 5).

**Table 5:** The activity level of caspase-3 in polycystic kidney epithelial cells

	Control	2-DG	MET	2-DG+MET
12 h	1.00 ± 0.04	1.01 ± 0.04	1.00 ± 0.04	1.14 ± 0.02***
24 h	1.00 ± 0.05	1.02 ± 0.03	1.00 ± 0.04	1.13 ± 0.03***
36 h	1.00 ± 0.06	1.21 ± 0.07***	1.00 ± 0.04	1.38 ± 0.09***
48 h	1.00 ± 0.03	1.35 ± 0.04***	1.00 ± 0.06	1.57 ± 0.03***

*Control:* normal group, *2-DG* 2-deoxyglucose-alone group, *MET* metformin alone group, *2-DG+MET* the combination of 2-DG and metformin

***p < 0.001 vs Control

### Effects of 2-DG or MET alone and combination on glucose metabolic phenotypes in human polycystic kidney epithelial cells

**Fig. 6:**
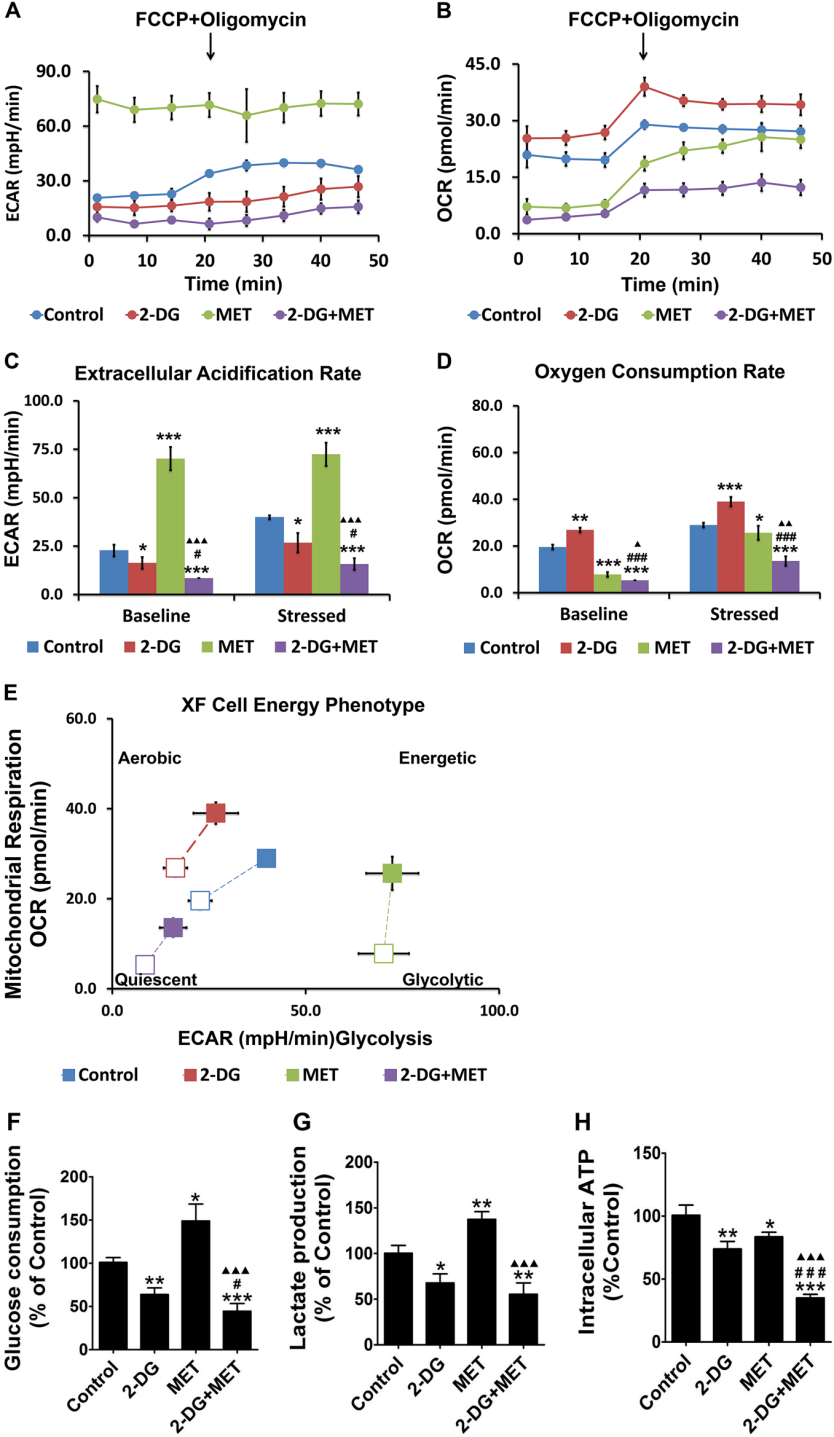
Effects of 2-deoxyglucose (2-DG) and metformin (MET) on the metabolism of WT9-7 cells. **a**, **b** The real-time mitochondrial bioenergetic profiles and glycolytic profiles were generated using the Seahorse XF96 Analyzer. **c**, **d** Quantitative analysis of the total baseline and stressed bioenergetic profiles for the oxygen consumption rate (OCR) and extracellular acidification rate (ECAR) of the four groups of WT9-7 cells; *p < 0.05,**p < 0.01, ***p < 0.001 vs Control, ^#^*p* < 0.05, ^##^*p* < 0.01, ^###^*p* < 0.001 vs 2-DG, ^▲^*p* < 0.05, ^▲▲^*p* < 0.01, ^▲▲▲^*p* < 0.001 vs MET. **e** Cell energy phenotypes were generated using the Seahorse XF96 Analyzer. **f** WT9-7 cells were treated with 5 mM 2-DG and 2.5 mM MET for 48 h and their glucose uptake was measured; *p < 0.05, **p < 0.01, ***p < 0.001 vs Control, ^#^*p* < 0.05, ^##^*p* < 0.01, ^###^*p* < 0.001 vs 2-DG, ^▲^*p* < 0.05, ^▲▲^*p* < 0.01, ^▲▲▲^*p* < 0.001 vs MET. **g** WT9-7 cells were treated with 5 mM 2-DG and 2.5 mM MET for 48 h and their lactate production was measured; *p < 0.05, **p < 0.01, ***p < 0.001 vs Control, ^#^*p* < 0.05, ^##^*p* < 0.01, ^###^*p* < 0.001 vs 2-DG, ^▲^*p* < 0.05, ^▲▲^*p* < 0.01, ^▲▲▲^*p* < 0.001 vs MET. **h** WT9-7 cells were treated with 5 mM 2-DG and 2.5 mM MET for 48 h and their ATP production was measured; *p < 0.05,**p < 0.01, ***p < 0.001 vs Control, ^#^*p* < 0.05, ^##^*p* < 0.01, ^###^*p* < 0.001 vs 2-DG, ^▲^*p* < 0.05, ^▲▲^*p* < 0.01, ^▲▲▲^*p* < 0.001 vs MET

Similar to tumor cells, PKD cyst epithelial cells also show the Warburg effect. In these cells, the glycolytic activity is significantly enhanced, and the intracellular ATP production is increased. Accordingly, the AMPK activity is significantly decreased, the mTOR activity is increased and the cell proliferation ability is enhanced. To investigate the effects of the combination of 2-DG and MET on glucose metabolism phenotype, we performed extracellular flux analysis on the cystic epithelial cells treated with 2-DG, MET and their combination using a Seahorse Extracellular Flux Analyzer. ECAR (extracellular acidification rate) represents the glycolysis level and OCR (oxygen consumption rate) represents the mitochondrial respiration level. We measured the real-time changes in ECAR and OCR under baseline (regular measurements, repeated 3 times) and stressed conditions (regular measurements, repeated 5 times) (Fig. 6a, b) to determine the two key parameters of cell energy metabolism: the baseline phenotype and the stressed phenotype.

First, we observed the effects of 2-DG, MET and their combination on the ECAR and OCR levels under baseline conditions in cystic epithelial cells. The results of quantitative analysis showed that compared with control group, 2-DG group alone had lower ECAR level (reflecting a decrease in the glycolysis level) (*p* < 0.05), whereas the OCR level increased. This finding indicates a compensatory increase in mitochondrial oxidative phosphorylation to supply sufficient energy to the cells. However, MET alone reduced the OCR level in cystic epithelial cells (reflecting a decrease in the oxidative phosphorylation level) (*p* < 0.001), whereas the ECAR increased. This finding indicates a compensatory increase in the cellular glycolytic capacity to supply sufficient energy to the cells (*p* < 0.001). When cystic epithelial cells were treated with 2-DG+MET, both the ECAR and OCR levels declined, suggesting that the combination of 2-DG and MET significantly decreased both the glycolysis and oxidative phosphorylation levels in the cells (*p* < 0.001) (Fig. 6c, d).

Next, we observed the effects of 2-DG, MET and their combination on the ECAR and OCR levels under stressed conditions in cystic epithelial cells. The stress factors include the metabolic modulator oligomycin, which inhibits mitochondrial ATP synthase and induces cellular glycolytic potential, and carbonyl cyanide-*p*-trifluoromethoxyphenylhydrazone (FCCP), which uncouples mitochondrial oxidative phosphorylation and induces maximal mitochondrial respiration^22^. The results of the quantitative analysis showed that compared with control group, 2-DG group alone had a lower ECAR level (reduced glycolytic capacity), whereas the OCR under stressed conditions increased in the cystic epithelial cells (*p* < 0.05). These findings indicate a compensatory increase in mitochondrial oxidative phosphorylation to supply sufficient energy to the cells. However, MET alone reduced the OCR level (reflecting a decrease in the oxidative phosphorylation level) (*p* < 0.001), whereas the ECAR level under stressed conditions increased in the cystic epithelial cells. These results indicate a compensatory increase in the cellular glycolytic capacity to supply sufficient energy to the cells (*p* < 0.001). When cystic epithelial cells were treated with 2-DG+MET, both the ECAR and OCR levels declined, suggesting that the combination of 2-DG and MET significantly lowered both the glycolysis and oxidative phosphorylation levels in the cells (Fig. 6c, d).

Figure 6e more clearly shows that regardless of whether the cells were in the baseline (open marks) or stressed (solid marks) condition, the ECAR and OCR levels were very low in the cystic epithelial cells in 2-DG+MET group. The values were located in the lower left corner of the coordinate system, indicating very low glycolysis and oxidative phosphorylation levels in these cells.

### Effects of 2-DG or MET alone and combination on glucose intake, lactate production and ATP concentration in human polycystic kidney epithelial cells

To further evaluate the effects of 2-DG and MET on glucose metabolism, we measured the glucose intake, lactate production and ATP concentration in cystic epithelial cells treated with 2-DG, MET and their combination. The results showed that both glucose intake and lactate production were decreased, while ATP synthesis was also significantly decreased in 2-DG group compared with control group. This finding indicates that 2-DG significantly decreased the glycolysis level in the cystic epithelial cells (Fig. 6f–h). Both glucose intake and lactate production were markedly increased, whereas ATP synthesis was markedly decreased in MET group compared with control group. This finding indicates that MET decreased the oxidative phosphorylation level in cystic epithelial cells, resulting in decreased ATP synthesis. However, a compensatory increase in the glycolysis level occurred, leading to an increase in lactate metabolites (Fig. 6f–h). The glucose intake and lactate production were decreased, while the ATP synthesis was also significantly reduced in 2-DG+MET group compared with control group. These findings further confirm that the combination of 2-DG and MET simultaneously decreased the metabolism levels of intracellular glycolysis and oxidative phosphorylation, thereby depleting the ATP supply of cells (Fig. 6f–h).

## Discussion

PKD is a hereditary disease caused by the mutations in PKD1 or PKD2 gene^23^. The abnormal proliferation of polycystic kidney epithelial cells is a main cause of the gradual enlargement of cysts. Except for symptomatic treatment, renal transplantation and dialysis for end-stage renal disease, there have been no clinically effective, targeted treatment drugs^24^.

A study in 2013 accidentally found that polycystic kidney cells mainly rely on aerobic glycolysis to produce a large quantity of ATP; this metabolic phenotype change is very similar to that in tumor cells. Therefore, inhibition of glycolysis may become a method to treat tumor and PKD. AMPK is a key molecule in the regulation of energy metabolism. mTOR not only regulates proliferation, but also plays an important role in regulating energy metabolism. AMPK can specifically inhibit mTOR activity^25^. A decreased AMPK activity and an increased mTOR activity were found in polycystic kidney cells^2^.

2-DG, the glucose analog, acts as a competitive inhibitor of the glycolytic enzyme hexokinase^26^. Upon transport into the cells, 2-DG is phosphorylated to 2DG-phosphate (2DG-P) by hexokinase. However, unlike glucose-6-phosphate (G-6-P), 2DG-P cannot be further metabolized by phosphohexose isomerase, which converts G-6-P to fructose-6-phosphate^27^. Thus, 2DG-P is trapped and accumulates in the cells, leading to inhibition of glycolysis mainly at the step of the phosphorylation of glucose by hexokinase. Inhibition of this rate-limiting step by 2-DG depletes the cellular ATP, leading to blockage of cell cycle progression and cell death in vitro^28^.

Our results showed that 2-DG could decrease the glycolysis level in polycystic kidney epithelial cells. The cellular intake of glucose, lactate production and intracellular ATP production were decreased, whereas the phosphorylated AMPKα level was increased by feedback in 2-DG group. Further, the activation levels of phosphorylated mTOR, p70 S6K and 4E-BP1 were downregulated in 2-DG group. Additionally, 2-DG increased the activation level of PI3K-Akt and indirectly inhibited the over-activation of B-Raf-ERK compared with that in control group. 2-DG also extended the G1/G0 phase of polycystic kidney epithelial cells. Although 2-DG alone can inhibit the proliferation of polycystic kidney epithelial cells, a high dose has side effects. Toxicity due to off-target effects has been attributed to this compound in clinical trials, and 3/10 mice in this study were killed early because of weight loss during 2-DG treatment^29^. Therefore, in this study, in order to decrease side effects, we selected small dose of 2-DG to observe its effect on the proliferation of polycystic kidney epithelial cells.

MET, as first-line hypoglycemic drug, has been used for treatment of type 2 diabetes and clinical trial of cancer treatment^30^. Our results showed that MET reduced intracellular ATP production by decreasing the mitochondrial oxidative phosphorylation level in polycystic kidney epithelial cells; MET can inhibit the proliferation of polycystic kidney epithelial cells by activating AMPKα activity (increase in the phosphorylated AMPKα level) and inhibiting mTOR pathway activity (decrease in the phosphorylated mTOR, p70 S6K and 4E-BP1). However, when oxidative phosphorylation metabolism was inhibited, there was a compensatory increase in the glycolysis level in the polycystic kidney epithelial cells; the cellular intake of glucose increased, and a large quantity of lactate was produced. Despite the good clinical tolerance of MET, lactic acidosis remains the primary side effect. Moreover, because MET is mainly cleared by the kidneys, chronic renal disease has been considered a potential predisposing factor for this complication^14^. Thus, treatment with MET alone has some limitations.

Given the limitations of 2-DG and MET, at present, many studies use their combination for the treatment of tumors^19,31^. Therefore, in this study, we investigated whether the combination of 2-DG and MET at small dose of level can more effectively inhibit the proliferation of polycystic kidney epithelial cells and lower the side effects of the two drugs. The results showed that their combination simultaneously inhibited glycolysis and oxidative phosphorylation metabolism in polycystic kidney epithelial cells, significantly decreased glucose intake, lactate production and ATP yield, and significantly decreased the activation levels of proliferation signaling pathway molecules, including mTOR, B-Raf-ERK and PKA. Moreover, we found that the combination of low-dose MET and 2-DG significantly upregulated the PI3K/Akt activation level, led to cell cycle arrest at G1/G0 phase and significantly inhibited cell proliferation. Inhibition of apoptosis has been shown to delay renal cyst growth in some animal models of PKD. In present study, either MET or 2-DG alone had little effect on the apoptosis of cystic epithelial cells, whereas their combination enhanced early apoptosis and increased caspase-3 activity in the cells.

In conclusion, this study presents the first evidence that the combinational use of low-dose 2-DG and MET can markedly inhibit cell proliferation via simultaneously inhibiting glycolysis and oxidative phosphorylation metabolism, reducing intracellular ATP production, activating AMPK activity, and thereby inhibiting the activation of the mTOR proliferation signaling pathway in polycystic kidney epithelial cells. Furthermore, the combination of these two drugs stimulates the activation level of PI3K/Akt, prevents the over-activation of B-Raf-ERK signaling pathway molecules and, finally, inhibits over-proliferation by arresting the cells at the G0/G1 phase. More importantly, combinational use of low-dose 2-DG and MET can lower the side effects of each drug. This study provides a new potential strategy for the future clinical use of these two drugs for the treatment of human PKD.

## Materials and methods

### Cell culture studies

The human autosomal dominant PKD cyst-lining epithelial cell line WT9-7 was purchased from American Type Culture Collection (ATCC, USA). Cells were seeded into Dulbecco’s modified Eagle’s medium (ATCC, USA) containing 10% fetal bovine serum (FBS; HyClone, Canada) and cultured in an incubator at 37 °C with 5% CO_2_. Cells were digested using 0.25% trypsin and subcultured at a ratio of 1:6 when 80% confluence was reached. The experimental groups were as follows: (1) control group: normal culture; (2) 2-DG group: adherent cells treated with different concentrations of 2-DG (MedChem Express, USA); (3) MET group: adherent cells treated with different concentrations of MET (Sigma, US); and (4) 2-DG+MET group: adherent cells treated with different concentrations of 2-DG and MET simultaneously. The HRPTEpiC were purchased from ScienCell Research Laboratories (ScienCell, USA). Cells were seeded into Epithelial Cell medium (ScienCell, USA) containing 2% FBS (HyClone, Canada) and cultured in an incubator at 37 °C with 5% CO_2_. Cells were digested using 0.25% trypsin and subcultured at a ratio of 1:2 when 90% confluence was reached. The experimental groups were as follows: (1) control group: normal culture; (2) 2-DG group: adherent cells treated with 5 mM concentration of 2-DG; (3) MET group: adherent cells treated with 2.5 mM concentration of MET; and (4) 2-DG+MET group: adherent cells treated with 5 mM 2-DG and 2.5 mM MET simultaneously.

### CCK-8 assay

After treatment with 2-DG, MET, and 2-DG+MET, the viability of WT9-7 human polycystic kidney epithelial cells were measured using a CCK-8 cell proliferation-cytotoxicity assay kit (Beyotime, China), and the optimal treatment concentration was selected. The polycystic kidney epithelial cell suspension was adjusted to a concentration of 3 × 10^7^/L and seeded in 96-well plates at 3000/well. The cells were synchronized in FBS-free medium for 12 h and then treated with different concentrations of 2-DG (0.6, 2.5, 10 and 40 mM), MET (0.6, 2.5, 10 and 40 mM) and 2-DG+MET (1.3 + 0.6, 2.5 + 1.3, 5 + 2.5 and 10 + 5 mM). Finally, the absorbance of polycystic kidney epithelial cell cultures at 450 nm was measured at 12, 24, 36 and 48 h of treatment with different drugs using a microplate reader (Bio-Rad, USA). The viability of HRPTEpiC was measured using a CCK-8 cell proliferation-cytotoxicity assay kit The human renal proximal tubular epithelial cells were adjusted to a concentration of 3 × 10^7^/L and seeded in 96-well plates at 3000/well. The cells were synchronized in FBS-free medium for 12 h and then treated with 2-DG (5 mM), MET (2.5 mM) and 2-DG+MET (5 and 2.5 mM). Finally, the absorbance of HRPTEpiC cells were measured at 48 h of treatment with different drugs using a microplate reader (Bio-Rad, USA). The percentage cell viability was calculated based on the absorbance of the drug-treated cells relative to the absorbance of the control cells. Experiments were performed in triplicate, and sextuplet wells were used.

### EdU proliferation assay

Cell proliferation was assayed using Click-iT® EdU Imaging kits (Invitrogen™ Thermo Fisher Scientific, USA). The EdU provided in the kit is a nucleoside analog of thymidine and is incorporated into DNA during active DNA synthesis. Detection is based on a click reaction, a copper-catalyzed covalent reaction between an azide and an alkyne. Briefly, WT9-7 polycystic kidney epithelial cells were seeded in 6-well plates at 10^5^/well, synchronized in FBS-free medium for 12 h, and then treated with 2-DG (5 mM), MET (2.5 mM) or 2-DG+MET (5 + 2.5 mM) for 36 h. Thereafter, 1:1000 EdU probe was added to the medium, and the cells were cultured for another 12 h and then fixed with 4% paraformaldehyde. Following the experimental procedure of the Click-iT® EdU Imaging kits, the nuclei were labeled with 4’,6-diamidino-2-phenylindole (DAPI, a fluorescent dye capable of binding strongly to DNA), and images were acquired by confocal laser microscopy.

### Cell cycle analysis

WT9-7 polycystic kidney epithelial cells were seeded in 25 cm^2^ culture flasks and synchronized in FBS-free medium for 12 h. The cells were incubated for another 48 h after the addition of 2-DG (5 mM), MET (2.5 mM) or 2-DG+MET (5 + 2.5 mM). The cells were washed twice with phosphate-buffered saline (PBS), suspended in 75% ethanol and fixed by incubation in 75% ethanol at 4 °C overnight. Fixed cells were collected by centrifugation, washed with PBS, treated with RNase (50 μg/mL; Sigma, USA) and stained with propidium iodide (PI; 50 μg/mL; Sigma, USA). A FACS flow cytometer (BD Co., USA) was used to determine the cellular DNA contents. The percentage of cells in the G0/G1, S and G2/M phases was determined using Cell FIT Cell Cycle Analysis software (version 2.01.2; BD).

### Caspase-3 activity assay

WT9-7 polycystic kidney epithelial cells were seeded in 25 cm^2^ culture flasks and treated with 2-DG (5 mM), MET (2.5 mM) or 2-DG+MET (5 + 2.5 mM) for 12, 24, 36 and 48 h. The proteins were collected, and the protein concentration in each group was measured by the bicinchoninic acid (BCA) assay. The protein concentration for each group was adjusted to the same level, and 50 μL of protein was taken for analysis. Caspase-3 activity was evaluated using a Caspase-3/CPP32 Colorimetric Assay kit (Biovision, USA), according to the manufacturer’s instructions. Finally, the absorbance of each sample at 405 nm was measured using a microplate reader. The percentage of caspase-3 activity was calculated as the absorbance of the drug-treated cells relative to the absorbance of the control vehicle-treated cells.

### Cell apoptosis evaluated using the annexin V-fluorescein isothiocyanate (FITC) assay

Cell apoptosis was evaluated using an Apoptosis Detecting kit (BD, USA) and analyzed by flow cytometry (FCM). Briefly, WT9-7 polycystic kidney epithelial cells were seeded in 25 cm^2^ culture flasks and treated with 2-DG (5 mM), MET (2.5 mM) or 2-DG+MET (5 + 2.5 mM) for 48 h. Following the manufacturer’s instructions, the cells were labeled with annexin V-FITC and PI. Samples were examined using FCM, and the results were analyzed using CellQuest software (Becton Dickinson, San Jose, CA).

### Assay of cell energy metabolism phenotypes

The Seahorse XF Cell Energy Phenotype Test in conjunction with a Seahorse Bioscience XF96 Extracellular Flux Analyzer measures the mitochondrial respiration and glycolysis under baseline and stressed conditions and provides the two key parameters of cell energy metabolism: the baseline phenotype and the stressed phenotype. Briefly, WT9-7 cells were plated at 1000 cells/well (~30% confluence) in a Seahorse XF Cell Culture Microplate (Seahorse Bioscience), and adherent cells were treated with 2-DG (5 mM), MET (2.5 mM) or 2-DG+MET (5 + 2.5 mM) for 48 h. Immediately prior to an experiment, the growth medium was removed from each well, the cells were rinsed with freshly prepared Seahorse assay medium (Seahorse Bioscience), and a final volume of 180 μL of assay medium was added to each well. The plate was incubated in a 37 °C incubator lacking CO_2_ for 1 h prior to the assay. Mitochondrial and glycolytic bioenergetic profiles were generated by taking baseline measurements of the OCR and ECAR of live cells in real time for three measurement cycles and then sequentially injecting oligomycin (inhibits ATP synthase, 1 μg/mL final concentration) and FCCP (uncouples mitochondrial oxidative phosphorylation and induces maximal respiration, 1 μg/mL final concentration) to measure the stressed OCR and ECAR for five measurement cycles.

### Measurement of intracellular glucose intake, extracellular lactate production and intracellular ATP synthesis

Polycystic kidney epithelial cells WT9-7 were seeded in 25 cm^2^ culture flasks and treated with 2-DG (5 mM), MET (2.5 mM) or 2-DG+MET (5 + 2.5 mM) for 48 h. The concentrations of glucose, lactate and ATP were determined using a Glucose Assay kit (Biovision, USA), Lactate Colorimetric/Fluorometric Assay kit (Biovision, USA) and CellTiter-Glo® 2.0 Luminescent Cell Viability Assay (Promega, USA), respectively, according to the manufacturer’s instructions.

### Western blot analysis

Proteins were extracted from WT9-7 cells using RIPA lysis buffer (50 mM Tris-HCl, pH 7.5, 150 mM NaCl, 0.5% deoxycholate, 1% Nonidet P-40, 0.1% SDS, 1 mM phenylmethylsulfonyl fluoride and protease cocktail at 1 μg/mL). The protein concentrations were determined using a BCA kit (Pierce, USA). Protein samples (50 μg per lane) were separated by 6%, 8%, 10% or 12% sodium dodecyl sufate–polyacrylamide gel electrophoresis (SDS-PAGE) electrophoresis and transferred to nitrocellulose (NC) membranes. After Ponceau S staining, the membranes were incubated overnight in 5% non-fat milk at 4 °C and then incubated with primary antibodies against p-AMPKα, p-mTOR, p-4E-BP1, p-p70 S6 kinase, p-PKA, p-PI3 kinase p85, p-Akt, p-B-Raf, p-MEK1/2, p-p44/42 MAPK (Erk1/2), PCNA, cyclin E1, Cdk2, Cdk4, (Cell Signaling Technology, USA), cyclin D3 (Wuhan Sanying Biotech Co., Ltd., China) and β-actin (Sigma, USA). Immunoreactive bands were visualized using ECL reagent (KeyGen Biotech Co., Ltd., China), according to the manufacturer’s instructions, and exposure to X-ray film. The protein band intensities were quantified using ImageJ software (NIH, Bethesda, USA).

### Statistical analyses

Student’s *T*-test was used to calculate statistical significance between two experimental groups, analysis of variance was used to calculate statistical significance between more than two experimental groups, and multivariate analysis using factorial analysis. Measurement data are expressed as the mean ± SEM. Statistical analyses were performed using the SPSS 19.0 software package (SPSS, Inc., USA). Values of *p* < 0.05 were considered statistically significant.
